# A national examination of discrimination, resilience, and depressive symptoms during the COVID-19 pandemic: the *All of Us* Research Program

**DOI:** 10.3389/fpsyg.2023.1175452

**Published:** 2023-09-26

**Authors:** Stephanie H. Cook, Erica P. Wood, Emma Risner, Chenziheng Allen Weng, Yao Xin

**Affiliations:** ^1^Department of Social and Behavioral Sciences, New York University School of Global Public Health, New York, NY, United States; ^2^Department of Biostatistics, New York University School of Global Public Health, New York, NY, United States

**Keywords:** discrimination, mental health, COVID-19, resilience, linear mixed modeling

## Abstract

**Objective:**

To examine the impact of resilience on the association between discrimination and trajectories of depressive symptoms during the COVID-19 pandemic across racial and ethnic groups.

**Methods:**

Data were drawn from 5 waves of the All of Us Research Program’s survey on the impact of COVID-19 on the lives of American adults. Linear mixed-effects models were fitted to assess the association between discrimination exposure throughout the pandemic and depressive symptoms over time. An interaction term was introduced between resilience and discrimination exposure to assess if resilience buffered the association between discrimination and depressive symptoms over time. Race-stratified linear mixed-effects models examined racial/ethnic differences in the association between resilience, discrimination, and depressive symptoms over time.

**Results:**

Fifty-one thousand nine hundred fifty-eight participants completed surveys between May and December of 2020. Results indicated that exposure to more discrimination was associated with increasing trajectories of depressive symptoms over time (*b* = 0.48, *p* < 0.001). However, resilience moderated the association between discrimination and well-being over time such that higher resilience mitigated the detrimental effect of experiencing discrimination on depressive symptoms across time (*b* = −0.02, *p* < 0.001).

**Conclusion:**

Identifying protective features such as resilience can promote the development of culturally tailored interventions to address mental health in the context of discrimination.

## 1. Introduction

Great disparities in morbidity and mortality have been discovered during the COVID-19 pandemic (Khanijahani et al., 2021). Indeed, a recent systematic review found that racial/ethnic minorities (e.g., Black and Latinx individuals) have a higher risk of COVID-19 infection as well as mortality related to the infection as compared to non-Latinx white individuals (Khanijahani et al., 2021). However, there continues to be much debate about the drivers of these disparities (Alcendor, 2020; Chowkwanyun and Reed, 2020; Dalsania et al., 2022). For instance, Alcendor (2020) found that risk factors such as hypertension influenced patterns of COVID-19 morbidity and mortality among racial/ethnic populations. Further, Dalsania et al. (2022) examined how social determinants of health (e.g., poverty, insurance rates) influenced COVID-19 mortality and found that countries with poorer social conditions had higher levels of COVID-19 mortality as compared to countries with better social conditions. However, one social determinant of health that has been understudied in this context is exposure to discrimination despite research demonstrating an increase in discrimination against racial/ethnic minorities (e.g., Asian-Americans) since the start of the COVID-19 pandemic (Elias et al., 2021). Further, less is known about how the COVID-19 pandemic has impacted trajectories of mental health and well-being among different racial/ethnic groups, and it remains unclear the degree to which discrimination is associated with changes in well-being throughout the pandemic. Thus, we aim to examine trajectories of racial discrimination and well-being across the COVID-19 pandemic across racial and ethnic identities.

### 1.1. Discrimination and trajectories of depressive symptoms

There is a plethora of research suggesting that exposure to discrimination is negatively associated with depressive symptoms (Priest et al., 2013). Further, throughout the COVID-19 pandemic, there has also been increased visibility to raced-based inequities in the United States (Chowkwanyun and Reed, 2020). Indeed, during this time, there were reported increases in discriminatory language and attacks particularly targeted toward Asian American as well as other racial/ethnic minority groups (Andrade et al., 2020; Chowkwanyun and Reed, 2020; Gao and Liu, 2020). Further, at the same time, heightened racial tension was observed throughout the United States as several cases of police violence against Black Americans came to light and led to country-wide protests against systemic racism and inequity (Galea and Abdalla, 2020). Overall, research has found that these increases in racial discrimination during the COVID-19 pandemic was associated with poorer mental health (Jaspal and Lopes, 2021; Maleku et al., 2021; Wu et al., 2021), particularly among Asian and Black Americans (Czeisler et al., 2020; Chae et al., 2021; McKnight-Eily et al., 2021). What is less known, however, is how changes in exposure to discrimination are associated with trajectories of depressive symptoms during the COVID-19 pandemic. The minority stress model posits that the accumulation of minority stressors over time, in this case discrimination, eventually culminates in poorer mental health (Brooks, 1981; Meyer, 2003). However, during such an acute time of unrest, and more specifically, during a pandemic, it is unclear how experiences of discrimination may be associated with trajectories of depressive symptoms across different racial/ethnic groups.

### 1.2. Discrimination, resilience, and depressive symptoms

Resilience theory conceptualizes the ways in which individuals thrive in the face of adversity (Ong et al., 2006). Resilience encapsulates the social, emotional, and individual-level factors that influence the ways in which individuals respond and adapt to stress (Zimmerman, 2013). There are three types of resilience that are posited to be factored into an individual’s overall wellbeing: trait, outcome, and process resilience (Ong et al., 2006; Hu et al., 2015; Linnemann et al., 2022). Trait resilience is the idea that people have a baseline level of resilience, and this baseline resilience helps them to navigate difficult situations, regardless of the severity of the situation (Hu et al., 2015). On the other hand, outcome resilience refers to the process of adapting and responding to stress using a variety of resources (e.g., economic resources, social resources) while sustaining positive mental health (Linnemann et al., 2022). If outcome resilience is observed over a sustained period of time, then the progression that underpins resilience is considered to be a process (i.e., “process resilience”; Linnemann et al., 2022). However, in this investigation, we focus on trait resilience given the wide disruption experienced in social and economic resources during the early COVID-19 pandemic. Trait resilience has been positively associated with mental health outcomes. For instance, in a systematic review of the literature, Hu et al. (2015) found that higher levels of trait resilience was generally associated with better overall mental health (e.g., lower depressive symptoms). In addition, in terms of discrimination and mental health, research has found that resilience buffers the association between discrimination and depression such that those who are more resilient experience better mental health outcomes in the face of discrimination in comparison to those who are less resilient (Yoon et al., 2019). Thus, in terms of the COVID-19 pandemic, resilience theory suggests that those who are more resilient will have better mental health in the face of discrimination than individuals who are less resilient. However, it remains unclear how resilience may impact the ways in which discrimination may impact trajectories of depressive symptoms. For instance, do individuals high on discrimination and high in resilience decrease in their depressive symptoms at a slower rate as compared to individuals who are low in resilience and who experience discrimination events?

### 1.3. Racial and ethnic differences

The association between discrimination and trajectories of depressive symptoms may be moderated not only by resilience but also by race/ethnicity. Indeed, there is a large body of work demonstrating the detrimental impact of racial discrimination on mental health outcomes among racial/ethnic minorities (Carter et al., 2017). Further, researchers have found that there may be differences in the association between experiences of discrimination and depression by race/ethnicity due to the myriad experiences of different racial/ethnic groups (Carter et al., 2017). For instance, some research suggests that Black Americans are more likely to experience racial discrimination as compared to other racial/ethnic groups such as Asian Americans and white individuals (Ayalon and Gum, 2011). However, some research suggests that there may be differences in the strength of the relationship between discrimination and mental health among Black Americans and white Americans such that there is a stronger association between discrimination and mental health among white Americans as compared to Black Americans (Ayalon and Gum, 2011). However, differences in the association between discrimination, depressive symptoms, and resilience among different racial/ethnic groups is, at present, poorly understood. Thus, in the current investigation, we seek to examine the ways in which discrimination is associated with trajectories of depressive symptoms specifically in the time of COVID-19 where reported discrimination events increased among several racial and ethnic groups. Moreover, we also seek to understand how the perception of discrimination during a pandemic may differentially influence trajectories of depressive symptoms among different racial/ethnic groups. We hypothesize that discrimination will be associated with depressive symptoms throughout the COVID-19 pandemic such that those who experience more discrimination will have increasing trajectories of depressive symptoms over time. In addition, we hypothesize that those high on discrimination will have higher baseline depressive symptoms and steeper positive trajectories of depressive symptoms over time as compared to those low on discrimination. However, we hypothesize that resilience will buffer this association such that those who have higher trait resilience will have slower increasing trajectories of depressive symptoms over time as compared to those lower on trait resilience. Further, based on previous literature, we hypothesize that the association between discrimination and depressive symptoms will be stronger among people of color (e.g., Black Americans) compared to white individuals but that this association will be buffered by resilience. In particular, we hypothesize that greater exposure to discrimination will be associated with positive trajectories of depressive symptoms over time particularly among people of color; however, this association will be attenuated among those who possess high levels of resilience.

## 2. Methods

Data for this study were gathered from the All of Us Research Program administered by the National Institutes of Health (NIH). The All of Us Research Program is an extensive precision medicine performance program with the goal of enrolling more than 1 million participants who reflect the diversity of the United States in order to understand health patterns among adult Americans aged 18 and older (All of Us Research Program Investigators, 2019). The All of Us Research Program currently includes data on more than 430,000 participants, from a variety of sources, including questionnaires, electronic health record (EHR) information, and physical measurements.

Given the impact of the coronavirus disease 2019 (COVID-19), the All of Us Research Program implemented the COVID-19 Participant Experience (COPE) survey to better understand the impact of the pandemic on people’s lives, especially on mental health (Harris, 2020). The COPE survey began in May 2020 and concluded in February 2021. The COPE project included an online survey that consisted of questions pertaining to COVID-19 symptoms, social distancing, mental health, physical health, and coping mechanisms. The COPE surveys were administered across six time points (May 2020, June 2020, July 2020, November 2020, and December 2020, February 2021). The discrimination measure was not included in the February 2021 dataset and, thus, the current analysis utilizes the first 5 waves of data (May to December 2020). This secondary analysis was considered exempt from the New York University Institutional Review Board.

### 2.1. Study sample

The sample was drawn from participants who participated in All of Us Research Program’s COPE study from May to December 2020. The COPE survey was administered to a subset of participants who completed the baseline All of Us Research Program survey. The COPE survey was administered online and took an average of 20–30 min to complete, and included questions related to mental and physical health in relation to COVID-19 as well as questions pertaining to discrimination. To be included in the sample, participants had to have responded to at least one of the five COPE surveys. Of 64,690 participants who completed at least one COPE survey during the specific time period, 51,958 participants who responded to the baseline demographic survey and who had data pertaining to mental health, resilience, and discrimination experiences in at least one COPE survey were included in the present analyses.

### 2.2. Measures

#### 2.2.1. Outcome

##### 2.2.1.1. Depressive symptoms

Depressive symptom severity was assessed using the 9-item Patient Heal Questionnaire (PHQ-9; Kroenke et al., 2001). An example item is “Over the last 2 weeks, how often have you been bothered by little interest or pleasure in doing things?” Responses range from 0 = Not at all to 3 = Nearly every day. Responses were averaged with higher scores indicating an increase in depressive symptom severity. The Cronbach’s alpha was 0.88.

#### 2.2.2. Predictor

##### 2.2.2.1. Discrimination

Exposure to discrimination was ascertained using the 10-item Everyday Discrimination Scale (Williams et al., 1997). An example item is “In your day-to-day life, how often did this happen to you during the past month? You are treated with less courtesy than other people are.” Responses ranged on a 4-point Likert scale ranging from 1 = Never to 4 = Almost every day. If participants indicated on any item that they experienced discrimination, they were asked what the main reason is for these experiences (e.g., rage, age, religion, etc.). Responses were averaged, with higher scores indicating greater exposure to discrimination. The Cronbach’s alpha was 0.86.

#### 2.2.3. Moderator

##### 2.2.3.1. Resilience

Resilience to adapt to stress was measured with the Brief Resilient Coping Scale (BRCS; Sinclair and Wallston, 2004). The BRCS is a 4-item measure that examines how individuals cope with stress. Participants were asked to think about their behavior and actions in the past month, and an example item includes “I look for creative ways to alter difficult situations.” Responses ranged on a 5-point Likert scale from 1 = Does not describe me at all to 5 = Describes me very well. Responses were averaged, with higher scores indicating greater resilience. The Cronbach’s alpha was 0.76.

#### 2.2.4. Demographics and covariates

Demographic variables included age, gender identity, sexual orientation, race/ethnicity, income, and education. Age was calculated based on the participant’s birthdate and the date of the survey. Gender identity categories included “male” “female” “transgender” and “other.” Sexual orientation categories included “heterosexual,” “gay” or “lesbian,” “bisexual,” and “other.” Race/ethnicity was categorized from baseline demographic data into “White,” “Black,” “Asian,” “Latinx,” and “Other” racial and ethnic categories. Annual household income was categorized as “below $25 k” “$25 – $50 k,” “$50 – $75 k,” and “$75 k and above.” Highest level of education completed was categorized as “High school diploma or GED,” “College, 1–3 years,” and “College degree and above.”

##### 2.2.4.1. Social support

Social support was ascertained using 10 items from the RAND Medical Outcomes Study (MOS) Social Support Survey Instrument (Sherbourne and Stewart, 1991). Participants were asked to reflect upon their experiences with social support in the past month. An example item includes, “Choose the answer that best describes how often you can find this kind of support in the past month. Someone to help if you were confined to bed.” Responses ranged on a 5-point Likert ranging from 1 = None of the time to 5 = All of the time. Responses were averaged and higher scores on the social support scale denote more social support. The Cronbach’s alpha was 0.95.

##### 2.2.4.2. Loneliness

Loneliness was measured using 8 items from the UCLA Loneliness Scale (Russell et al., 1978). Participants were asked to reflect upon their experiences in the last month, with an example item being “I am unhappy being so withdrawn.” Responses ranged on a 4-Point Likert scale from 1 = Never to 4 = Often. Responses were averaged, and higher scores on the loneliness scale denote more loneliness. The Cronbach’s alpha was 0.86 after reverse scaling negatively correlated items.

##### 2.2.4.3. COVID-related impact

COVID-19 related impact was measured using 6 items developed for the COPE questionnaire that ascertain the impact of the COVID-19 pandemic on one’s day-to-day life (Chan et al., 2020). Participants were asked to reflect upon their experience over the last week, and an example item includes “In the past 7 days, I had trouble concentrating.” Responses ranged on a 5-point Likert ranging from 1 = Not at all to 5 = Extremely. Scores were averaged across the six items, with greater scores indicating more COVID-19 related impact. The Cronbach’s alpha was 0.83.

### 2.3. Analytic plan

We first examined descriptive statistics and bivariate correlations for each of the study variables. For bivariate analyses correlations were calculated between each wave of depressive symptoms and all variables of interest, the Pearson product moment correlations were calculated for continuous covariates, the Spearmen rank-order correlations were calculated for ordinal categorical covariates, and Analysis of Variance (ANOVA) was used for nominal categorical covariates.

In order to account for the nested nature of the longitudinal data, linear mixed effects modeling was utilized to examine our study hypotheses. First, we specified a fully unconditional model (Model 1) with no predictors to examine the amount of variation in depressive symptoms across individuals. We followed this model with a conditional model that incorporated both discrimination and a linear growth parameter (Model 2) wherein intercepts were allowed to vary across individuals. We then added relevant time-varying and time invariant covariates to our model (Model 3). In Model 4, we included an interaction term between resilience and discrimination exposure to examine if resilience moderated the association between exposure to everyday discrimination and depressive symptoms. Social support, loneliness, and COVID-19 impact were treated as time-varying covariates, whereas others race, gender, sexual orientation, age, income, and education were treated as time invariant. To examine our second hypothesis regarding racial differences, we ran a total of six moderation models in order to examine differences in the association between resilience, discrimination, and trajectories of depressive symptoms among white, Black, Latinx, Asian, Multiracial, and Other racial/ethnic identified individuals. We then examined the simple slopes and contrasts in slopes of the race-stratified models to examine differences in the main effect of discrimination on depressive symptoms and to examine differences in the moderating effect of resilience in the association between discrimination and depressive symptsom over time across racial and ethnic groups. All analyses were conducted using RStudio.

## 3. Results

### 3.1. Descriptive and bivariate statistics

Table 1 displays the sociodemographic characteristics of the study sample (*n* = 51,958). Participants were a mean age of 56.55 (SD = 15.97; median = 60; Table 1). The majority of the participants identified as cisgender female (64.47%), while the remaining identified as cisgender male (34.56%) or transgender/another gender identity (0.97%). Eighty seven percent of the participants identified as white, while the remaining identified as Black (5.21%), Asian (3.05%), Latinx (2.01%), multiracial (1.72%), or another racial/ethnic identity (1.25%). Moreover, the majority of participants reported having an annual income of $75,000 or more (57.28%) while the remaining reported an income less than $25,000 (10.36%), between $25,000 and $50,000 (15.81%), or between $50,000 to $75,000 (16.34%). In terms of education, the majority of participants reported having an advanced degree (40.48%) or a college degree (32.05%). The remaining participants reported less than high school education (0.50%), having a high school diploma or GED (5.86%), or having gone to college for 1–3 years (21.10%). Lastly, with respect to sexual orientation the majority of participants identified as heterosexual (89.91%), whereas the remaining identified as lesbian or gay (4.60%), bisexual (3.93%), or as asexual (1.56%).

**Table 1:** Demographic characteristics and covariate distributions in the estimation sample (*N* = 51,958).

		*N* ^a^	%^a^
Mean age (range), SD		56.55 (18–115)	15.97
Gender	Male	17,957	34.56
	Female	33,499	64.47
	Transgender/Other	502	0.97
Race	White	45,078	87.76
	Black	2,705	5.21
	Asian	1,587	3.05
	Latinx	1,042	2.01
	More than one race	896	1.72
	Other	651	1.25
Income	Less than 25 k	5,385	10.36
	25–50 k	8,214	15.81
	50–75 k	8,491	16.34
	75 k or greater	29,868	57.28
Sexual orientation	Straight	46,715	89.91
	Lesbian or gay	2,391	4.60
	Bisexual	2042	3.93
	None	810	1.56
Education	Less than high school	260	0.50
	12th grade or GED	3,047	5.86
	College 1–3 years	10,962	21.10
	College graduate	16,654	32.05
	Advanced degree	21,035	40.48
Mean resilience at baseline (range), SD		14.95 (4–20)	2.68
Mean PHQ9 (range), SD	Wave 1	4.97 (0–27)	5.11
	Wave 2	4.69 (0–27)	4.99
	Wave 3	4.68 (0–27)	4.99
	Wave 4	5.08 (0–27)	5.22
	Wave 5	5.29 (0–27)	5.44
Mean social support (range), SD	Wave 1	36.69 (10–50)	10.22
	Wave 2	39.58 (10–50)	10.25
	Wave 3	39.66 (10–50)	10.16
	Wave 4	39.51 (10–50)	10.04
	Wave 5	39.06 (10–50)	10.34
Mean Loneliness (range), SD	Wave 1	7.68 (0–24)	5.18
	Wave 2	7.44 (0–24)	5.25
	Wave 3	7.56 (0–24)	5.37
	Wave 4	7.62 (0–24)	5.34
	Wave 5	7.73 (0–22)	5.35
Mean COVID-related impact score (range), SD	Wave 1	8.50 (0–24)	4.85
	Wave 2	7.31 (0–24)	4.65
	Wave 3	7.57 (0–24)	4.73
	Wave 4	7.70 (0–24)	4.77
	Wave 5	7.26 (0–24)	4.79
Mean discrimination (range), SD	Wave 1	1.99 (0–27)	3.19
	Wave 2	1.93 (0–27)	3.20
	Wave 3	1.95 (0–27)	3.25
	Wave 4	2.38 (0–27)	3.62
	Wave 5	2.42 (0–25)	3.60

^a^Unless otherwise specified.

As shown in Table 1, mean depressive symptoms for waves 1–5 was 4.97, 4.69, 4.68, 5.08 and 5.29, respectively (range = 0 to 27). Moreover, the mean discrimination score was 1.99, 1.93, 1.95, 2.38, and 2.42 for waves 1–5, respectively (range = 0–27). Means and standard deviations for resilience, social support, loneliness, COVID-related impact are also presented in Table 1. Correlations between the depressive symptoms of each wave with covariates included in the model are presented in Table 2. Age, income, education, gender, Race/ethnicity, and sexual orientation were significantly correlated with each wave of mental depressive symptoms (*p* < 0.01; Table 2). Significant correlations were also observed between each wave of depressive symptoms and the baseline resilience score as well as between each wave of social support, loneliness, COVID-related impact score, and perceived discrimination (*p* < 0.001 for all pairs presented in Table 2). For depressive symptoms alone, the earlier waves of depressive symptoms correlated significantly to the later waves (wave 1 correlated with waves 2–5 and wave 2 correlated with waves 3–5, *p* < 0.001; Table 2).

**Table 2:** Correlations between variables and PHQ9 at each wave, in the estimation sample (*N* = 51,958).

	*N*	PHQ9				
		Wave 1	Wave 2	Wave 3	Wave 4	Wave 5
Age (baseline)		−0.32***	−0.30***	−0.31***	−0.32***	−0.31***
Resilience (baseline)	51,958	0.45***	0.43***	0.44***	0.43***	0.41***
Gender^1^		523.8***	363.5***	344.9***	61.87***	48.37***
Race^1^		24.93***	16.87***	12.41***	5.68***	3.26**
Sexual Orientation^1^		372.7***	245.3***	238.1***	53.18***	53.48***
Income^2^		−0.17***	−0.18***	−0.18***	−0.20***	−0.21***
Education^2^		−0.10***	−0.09***	−0.09***	−0.12***	−0.13***
PHQ9 (wave1)	31,198	1				
PHQ9 (wave2)	24,383	0.83***	1			
PHQ9 (wave3)	21,611	0.79***	0.83***	1		
PHQ9 (wave4)	4,534	0.77***	0.79***	-	1	
PHQ9 (wave5)	3,265	0.74***	0.78***	-	-	1
Social support (wave 1)	31,198	−0.32***	−0.29***	−0.30***	−0.30***	−0.25***
Social support (wave 2)	24,383	−0.33***	−0.33***	−0.32***	−0.34***	−0.30***
Social support (wave 3)	21,611	−0.33***	−0.33***	−0.36***	-	-
Social support (wave 4)	4,534	−0.35***	−0.34***	-	−0.33***	-
Social support (wave 5)	3,265	−0.31***	−0.30***	-	-	−0.33***
Loneliness (wave 1)	31,198	0.60***	0.55***	0.53***	0.55***	0.53***
Loneliness (wave 2)	24,383	0.56***	0.60***	0.56***	0.60***	0.54***
Loneliness (wave 3)	21,611	0.55***	0.57***	0.61***	–	–
Loneliness (wave 4)	4,534	0.56***	0.58***	–	0.63***	–
Loneliness (wave 5)	3,265	0.58***	0.57***	–	–	0.62***
Impact score (wave 1)	31,198	0.50***	0.44***	0.42***	0.42***	0.39***
Impact score (wave 2)	24,383	0.47***	0.51***	0.47***	0.47***	0.42***
Impact score (wave 3)	21,611	0.47***	0.48***	0.51***	–	–
Impact score (wave 4)	4,534	0.43***	0.44***	–	−0.36***	–
Impact score (wave 5)	3,256	0.43***	0.47***	–	–	0.48***
Discrimination (wave 1)	31,198	0.42***	0.40***	0.38***	0.39***	0.45***
Discrimination (wave 2)	24,383	0.39***	0.41***	0.40***	0.41***	0.37***
Discrimination (wave 3)	21,611	0.36***	0.36***	0.41***	–	–
Discrimination (wave 4)	4,534	0.41***	0.43***	–	0.44***	–
Discrimination (wave 5)	3,256	0.42***	0.39***	–	–	0.46***

All the correlations are Pearson’s correlations. The missing Pearson correlation values are due to insufficient sample sizes. Sample sizes for the presented correlations between time-varying covariates and mental well-being varied, with the biggest at 31,067 between wave 1 and wave 1, the smallest at 547 between wave 2 and wave 5. *p < 0.05, **p < 0.01, ***p < 0.001. ^1^ANOVA: *F* value, ^2^Spearmen’s Correlation: rho, *p*: * < 0.05, ** < 0.01, *** < 0.001.

### 3.2. Growth models

Table 3, Model 1 shows the results of the fully unconditional mixed-effects model. The results indicated that the overall mean of the PHQ-9 was 4.92 (standard error [SE] = 0.02) and varied significantly between individuals (*p* < 0.001). Model 2 demonstrated that PHQ-9 increased over time (*b* = 4.14, *p* < 0.001; Table 3, Model 2). Further, increased exposure to discrimination was associated with increasing trajectories of depressive symptoms across the five waves (*b* = 0.48, *p* < 0.001; Table 3, Model 2). Model fit increased when comparing model 1 (the fully unconditional model) with model 2 which included the effect of both of time and discrimination.

**Table 3:** Effect of discrimination on general well-being across five COPE waves (*N* = 51,958).

Variables			Model 1			Model 2			Model 3			Model 4		
			*β*	SE	*p*	*β*	SE	*p*	*Β*	SE	*p*	*β*	SE	*p*
Fixed effects														
Intercept			4.92	0.02	<0.001	4.14	0.03	<0.001	7.10	0.25	<0.001	6.38	0.26	<0.001
Time (survey waves)			–	–	–	−0.10	0.01	<0.001	0.05	0.01	<0.001	0.05	0.01	<0.001
Time-invariant	Race (ref: White)	Black	–	–	–	–	–	–	−1.13	0.07	<0.001	−1.10	0.07	<0.001
		Asian	–	–	–	–	–	–	−0.88	0.09	<0.001	−0.86	0.09	<0.001
		Latinx	–	–	–	–	–	–	0.23	0.11	0.03	0.24	0.11	0.03
		More than one race	–	–	–	–	–	–	0.18	0.11	0.12	0.19	0.11	0.10
		Other	–	–	–	–	–	–	0.08	0.13	0.53	0.08	0.13	0.08
	Gender (ref: female)	Male	–	–	–	–	–	–	−0.34	0.03	<0.001	−0.34	0.03	<0.001
		Transgender/Other	–	–	–	–	–	–	0.66	0.16	<0.001	0.67	0.16	<0.001
	Sexual orientation (ref: Straight)	Lesbian or Gay	–	–	–	–	–	–	0.39	0.07	<0.001	0.40	0.07	<0.001
		Bisexual	–	–	–	–	–	–	1.03	0.08	<0.001	1.03	0.08	<0.001
		None	–	–	–	–	–	–	0.94	0.13	<0.001	0.94	0.13	<0.001
	Age		–	–	–	–	–	–	−0.04	0.001	<0.001	−0.04	0.001	<0.001
	Income (ref: less than 25 k)	25–50 k	–	–	–	–	–	–	−0.58	0.06	<0.001	−0.57	0.06	<0.001
		50–75 k	–	–	–	–	–	–	−0.90	0.06	<0.001	−0.90	0.06	<0.001
		75 k and above	–	–	–	–	–	–	−0.99	0.006	<0.001	−0.98	0.06	<0.001
	Education (ref: less than HS)	Twelve or GED	–	–	–	–	–	–	−0.63	0.22	0.005	−0.54	0.22	0.02
		College 1–3	–	–	–	–	–	–	−0.58	0.22	0.007	−0.49	0.21	0.03
		College graduate	–	–	–	–	–	–	−1.00	0.22	<0.001	−0.91	0.21	<0.001
		Advanced degree	–	–	–	–	–	–	−1.04	0.22	<0.001	−0.95	0.22	<0.001
	Resilience (baseline)		–	–	–	–	–	–	−0.23	0.01	<0.001	−0.19	0.01	<0.001
Time-varying	Loneliness		–	–	–	–	–	–	0.34	0.003	<0.001	0.34	0.003	<0.001
	COVID-related impact		–	–	–	–	–	–	0.28	0.003	<0.001	0.28	0.003	<0.001
	Social Support		–	–	–	–	–	–	−0.002	0.002	0.16	−0.002	0.002	0.20
Exposure	Discrimination score (time-varying)		–	–	–	0.48	0.005	<0.001	0.20	0.004	<0.001	0.44	0.02	<0.001
Interaction	Discrimination × Resilience (baseline)		–	–	–	–	–	–	–	–	–	−0.02	0.001	<0.001
Random effects:														
Residual			4.71	2.17		4.88	2.21		4.22	2.06		4.23	2.06	
μ0j			21.47	4.63		16.99	4.12		8.16	2.86		8.10	2.85	
													
Fit:														
AIC			479,743.3			470,908.0			434,701.60			434,508.0		
Log likelihood			−239,686.7			−235,449.0			−217,323.80			−217,226.0		

Table 3, Model 3 displays the results of the adjusted conditional growth model with the inclusion of the relevant covariates. Results indicated that, as compared to white individuals, Black and Asian individuals had decreased depressive symptoms, on average, across time compared to white individuals (*b* = −1.13, *p* < 0.001 and *b* = −0.88, *p* < 0.001, respectively; Table 3, Model 3). On the other hand, Latinx individuals had increased depressive symptoms, on average, as compared to white individuals across time (*b* = 0.23, *p = 0.03*). Compared to cisgender females, those who identified as a cisgender male had decreased depressive symptoms, on average, across time (*b* = −0.34, *p* < 0.001). While those who identified as Transgender or other had increased depressive symptoms, on average, across time (*b* = 0.66, *p* < 0.001). Further, those who identified as lesbian/gay, bisexual, or who identified as having no sexual orientation had increased depressive symptoms, on average, as compared to heterosexuals across time (*b* = 0.39, *p* < 0.001; *b* = 1.03, *p* < 0.001; and *b* = 0.94, *p* < 0.001, respectively; Table 3, Model 3). In terms of income, those who made annual incomes between $25-$50 k, $50-$75 k and $75 k or above had decreased depressive symptoms as compared to those who made below $25 k (*b* = −0.58, *p* < 0.001, *b* = −0.90, *p* < 0.001, and *b* = −0.99, *p* < 0.001, respectively; Table 3, Model 3). With respect to education, those who had obtained a high school diploma or GED, went to college for 1–3 years, were college graduates, or earned advanced degrees had decreased depressive symptoms scores over time as compared to those who did not obtain a high school diploma (*b* = −0.63 *p* = 0.005, *b* = −0.58 *p* = 0.007, *b* = −1.00 *p* < 0.001 and *b* = −1.04, *p* < 0.001, respectively; Table 3, Model 3). Those who reported a higher resilience score at baseline were associated with decreased depressive symptoms on average compared to those who did not over time (*b* = −0.23, *p* < 0.001; Table 3, Model 3). With respect to loneliness, those who reported greater levels of loneliness experienced increased depressive symptoms on average, across time as compared to those who reported lower levels (*b* = 0.34, *p* < 0.001; Table 3, Model 3). Further, as compared to those who reported lower levels of COVID-19 impact, those who reported higher levels of COVID-19 impact had increasing trajectories of depressive symptoms over time (*b* = 0.28, *p* < 0.001; Table 3, Model 3). Lastly, those who reported experiencing more discrimination, as compared to those who reported experiencing less discrimination, had increasing trajectories of depressive symptoms over time (*b* = 0.20, *p* < 0.001; Table 3, Model 3).

Table 3, Model 4 shows the results of the multi-level model with the inclusion of the interaction term between discrimination and baseline resilience. The results suggest that baseline resilience moderated the association between discrimination and depressive symptoms over time (*b* = −0.02, *p* < 0.001; Table 2, Model 4), such that higher baseline resilience scores mitigated the detrimental effect of experiencing discrimination on depressive symptoms across time. We followed up the significant moderation analysis with an examination of simple slopes at −1 SD and below the mean, the mean, and +1 SD and above the mean for resilience (Preacher et al., 2006). We found that those who reported resilience scores within 1 SD of the mean (simple slope = 0.18, *p* < 0.001) and +1 SD and above the mean (simple slope = 0.14, *p* < 0.001) for resilience had slower increments in depressive symptoms over time with the same increase in discrimination as compared to those who were −1 SD and below the mean for resilience (simple slope = 0.23, *p* < 0.001). When comparing the simple slopes, there is a significant difference between each resilience groups (*p* < 0.001), with those with higher resilience having fewer depressive symptoms on average over time (Supplementary Table S2C).

**Figure 1:**
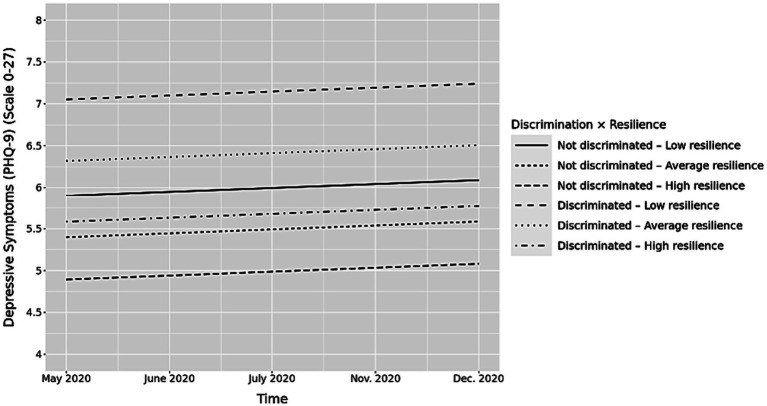
Longitudinal associations between discrimination and depressive symptoms by resilience score. *Y* axis starts at 4 to better reflect group differences.

A graphic presentation of the predicted depressive symptoms over time, controlling for covariates presented in Table 3, is shown in Figure 1. The majority of the estimation sample (60.93%) had no depressive symptoms (PHQ-9 0–4), followed by mild depressive symptoms (PHQ9 5–9; 24.28%), moderate depressive symptoms (PHQ-9 10–14; 9.46%), moderately severe depressive symptoms (PHQ-9 15–19; 3.24%), and severe depressive symptoms (PHQ-9 20–27; 2.08%) which are in line with population-based estimates (Rief et al., 2004; Kocalevent et al., 2013). While resilience and discrimination jointly predicted the intercept of depressive symptoms, resilience set the relative locations of the three pairs of groups. Within each of group, those experiencing discrimination all have decreased depressive symptoms across time. However, the gaps between the group that experienced discrimination and the group that did not experience discrimination differ by level of resilience (i.e., below the mean, at the mean, and above the mean of resilience). As Figure 1 shows, experiencing discrimination is associated with increased depressive symptoms among the low resilience group (i.e., 1 SD and below the mean). Indeed, there is a wider gap between levels of depressive symptoms among those who experienced discrimination and those who did not experience discrimination in the low resilience group indicating that those who experience discrimination and who possess low levels of resilience have increased depressive symptoms over time. On the other hand, there is a narrower gap between groups of individuals who experienced discrimination and who did not experience discrimination among the average (i.e., within 1 SD of the mean) and high (i.e., 1 SD and above the mean) resilience groups. This suggests that the interaction between resilience and discrimination is more pronounced among the low resilience group as compared to the average and high resilience groups.

### 3.3. Race-stratified analyses

Overall, we observed increased effects of discrimination on depressive symptoms among white (*b* = 0.21, *p* < 0.001; Supplementary Table S1A), Black, (*b* = 0.15, *p* < 0.001; Supplementary Table S1B), Asian (*b* = 0.19, *p* < 0.001; Supplementary Table S1C), Latinx (*b* = 0.18, *p* < 0.001; Supplementary Table S1D), multiracial (*b* = 0.14, *p* < 0.001; Supplementary Table S1E), and Other (*b* = 0.22, *p* < 0.001; Supplementary Table S1F) participants. When examining the moderating effect of resilience on the association between experiences of discrimination and general depressive symptoms over time, the effects of discrimination were mitigated with higher resilience scores among white (*b* = −0.02, *p* < 0.001; Supplementary Table S1A), Black (*b* = −0.01, *p* < 0.001; Supplementary Table S1B), and Asian (*b* = −0.02, *p* < 0.05; Supplementary Table S1C) individuals when controlling for relevant covariates (e.g., income, education, COVID-19 impact). However, the interaction between resilience and discrimination did not reach significance among Latinx, multiracial individuals and those who identified as Other.

**Figure 2:**
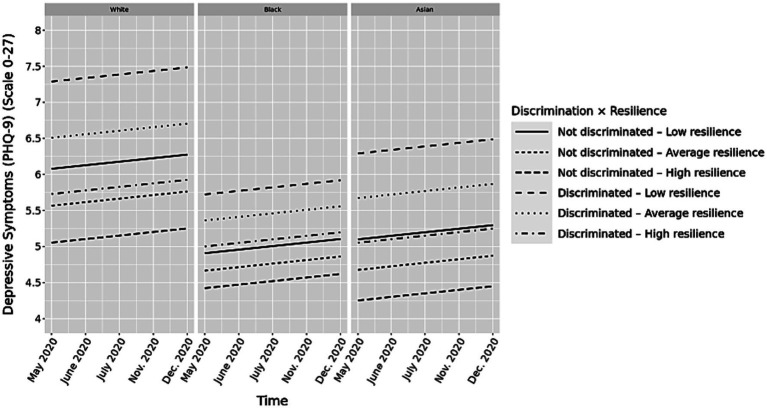
Longitudinal associations between discrimination, resilience score and depressive symptoms by race and ethnicity groups. *Y* axis starts at 4 to better reflect group differences.

Figure 2 displays a graphic representation of the moderating effect of resilience on the association between discrimination and general well-being for white, Black, and Asian individuals (controlling for covariates presented in Table 3). As in the pooled sample’s result, the different gaps between the groups with or without experiences of discrimination demonstrate that the buffering effect of resilience on the association between discrimination and depressive symptoms differed by race and ethnicity. Among white, Black and Asians with resilience −1 SD below the mean, the depressive symptoms increased the most over time (*b* = 0.21, *p* < 0.001; *b* = 0.16, *p* < 0.001; *b* = 0.23, *p* < 0.001; respectively) compared to those with resilience within 1 SD of the mean (*b* = 0.19, *p* < 0.001; *b* = 0.14, *p* < 0.001; *b* = 0.20, *p* < 0.001; respectively), and those with resilience +1 SD and above the mean (*b* = 0.13, *p* < 0.001; *b* = 0.12, *p* < 0.001; *b* = 0.16, *p* < 0.001; respectively). However, when contrasting simple slopes only, the difference in the mitigating effect of resilience on discrimination and depressive symptoms between white and Black individuals was found to be significant for resilience levels – 1 SD and below the mean and within 1 SD of the mean (*b* = 0.08, *p* < 0.001; *b* = 0.05, *p* = 0.001, respectively).

## 4. Discussion

The present study examined whether baseline levels of resilience, or one’s ability to use healthy coping mechanisms to successfully cope with experiences of stress, moderated the association between experiences of discrimination throughout the early COVID-19 pandemic and trajectories of depressive symptoms. Overall, our results supported our study hypothesis that resilience would attenuate the impact of discrimination on depressive symptoms over time. Across the whole study sample, we found that, on average, mean depressive symptoms decreased across the early COVID-19 pandemic. This finding is supported in previous literature, which broadly suggests that mental health symptoms decreased over the course of the COVID-19 pandemic which may be, in part, due to factors such as vaccine development and reduced social distancing restrictions (Fluharty et al., 2021). We also observed that baseline depressive symptoms differed by race and ethnicity such that those who identified as Latinx or multiracial had higher greater depressive symptoms, on average, as compared to white individuals. On the other hand, we observed that Black and Asian individuals had lower depressive symptoms, on average, than white individuals. We also observed a main effect of discrimination such that experiencing more discrimination was associated with increasing trajectories of depressive symptoms over time. A main effect for baseline resilience was also observed, which indicated that higher levels of baseline resilience were associated with lower levels of depressive symptoms over time. Further, we found that higher levels of baseline resilience attenuated the association between experiences of discrimination and depressive symptoms over time. Specifically, experiencing discrimination was associated with the highest levels of depressive symptoms across time among those low in baseline resilience (i.e., 1 SD and below the mean). Further, while those who experienced discrimination among the high (1 SD and above the mean) and average (within a SD of the mean) resilience group experienced higher levels of depressive symptoms compared to their respective resilience group that did not experience discrimination, depressive symptoms across time remained significantly lower compared to the low resilience group. This suggests that, overall, that resilience may buffer the negative effects of experiences of discrimination on depressive symptoms across time. Discussion of our findings as well as implications are described below.

Our findings suggesting the presence of a positive association between exposure to discrimination and depressive symptoms are well supported by the extant literature. For instance, one study used latent class analysis to examine trajectories of racial discrimination over time and resultant mental well-being across a cohort of 605 African American young adults spanning a total of 12 years (Lee et al., 2020). The authors found that those participants who experienced a moderate amount of racial discrimination over time had decreased mental well-being (e.g., depressive and anxiety symptoms) compared to those who experience a low amount of racial discrimination over time. Further, a meta-analysis examining racial/ethnic discrimination and well-being among adolescents found that increased perceptions of racial/ethnic discrimination were associated with decreased indicators of mental well-being, including depressive and anxiety symptoms, psychological distress, and externalizing behaviors (Benner et al., 2018). Further, in another meta-analytic review, it was found that perceived discrimination negatively impacted psychological well-being (e.g., depression, anxiety, psychological distress) and that the observed effect size was stronger among racial/ethnic minorities as compared to white individuals (Schmitt et al., 2014). Our findings add to the body of literature suggesting the negative impacts of discrimination on well-being specifically in the time of the early COVID-19 pandemic, a period of acute racial discrimination and xenophobia (Elias et al., 2021).

While our finding that discrimination is associated with increased depressive symptoms over time is well-supported in the overarching literature, we add to this body of work with our finding that higher levels of baseline resilience in the form of being able to adaptively cope with stress offset the negative effects of exposure to discrimination on depressive symptoms across the early COVID-19 pandemic. This finding is in line with previous research which suggests that possessing higher levels of resilience is associated with better overall mental health outcomes in the face of discrimination (Yoon et al., 2019). Emerging research conducted during the early COVID-19 pandemic has found similar findings. For instance, one study conducted with 242 Asian Americans found that increased instances of discrimination during COVID-19 was associated with increased resilience *via* increased social support behaviors which, in turn, was associated with increased subjective well-being (Yang et al., 2020). Our findings suggest that an individual’s ability to cope with stressors such as discrimination in a period of acute global stress (i.e., a pandemic) may play an important role in one’s mental health across time. It may be the case that an ability to cope with stress across multiple situations, such as the stress of dealing with the consequences associated with a pandemic as well as increasing levels of racial discrimination observed in relation to this pandemic, contributes significantly to one’s psychological adjustment over time. Further, there are varying types of racial discrimination (e.g., overt instances and microaggressions) which may have differential consequences for mental health and resilience processes (Hernández and Villodas, 2020; Loyd et al., 2022). Thus, an important area to consider in future research is examining methods through which to foster resilience across a variety of stressful situations. More specifically, research should examine *how* resilience is developed and maintained among individuals as well as across cultural identities. For instance, is it a personality trait that is developed over time or is it fostered through a series of mechanisms and experiences such as strong social support networks or other environmental and cultural features? Such research, in turn, could inform interventions that aim to improve mental health across individuals over time.

Our hypothesis regarding the role of race was not fully supported, we found that the interaction between baseline resilience and discrimination was significant for three of the six racial/ethnic groups (white, Black, Asian) whereas the interaction term did not reach significance within the models representing individuals who identified as Latinx, multiracial, or another racial/ethnic identity. These findings highlight that the appraisal of discrimination and the impact of resilience may differ in culturally meaningful ways that warrant further exploration, particularly among Latinx and individuals of multiracial identity. Indeed, previous research has found key cultural differences in the ways in which resilience mechanisms (e.g., ethnic identification) buffers the association between exposure to racial discrimination and mental health (Vines et al., 2017). One study examining the association between ethnic identity development, resilience to racial discrimination, and depressive symptoms among 125 ethnic minority adolescents (e.g., Mexican, Native) found that high ethnic affirmation (i.e., having positive feelings about one’s ethnic group) attenuated the association between stress experienced from discrimination and depressive symptoms (Romero et al., 2014). Thus, it may be that the way in which resilience was measured within our study did not wholly capture more salient processes of resilience such as positive ethnic identification among Latinx and multiracial individuals. Moreover, in another longitudinal study among 331 African American youth surveyed from age 16 to 18 found that high levels of experienced racial discrimination over time was associated with heightened allostatic load; however, this association disappeared among youth who received high emotional support from their parents and/or peers (Brody et al., 2014). Among Asian Americans, high levels of ethnic identity connectedness (Wei et al., 2012) as well as family support (Chae et al., 2012) have been shown to weaken the association between racial discrimination and poor mental health such as depressive symptoms. Further, a *post hoc* chi-square test for the effect of the interaction across these models indicated significant differences in the interaction between resilience and discrimination between the white and Other category whereas the difference between the Black and Other category was trending (see Supplementary Table S2B). While our Other category within this analysis comprised a wide range of identities (e.g., Native), these observed differences suggest that future research should aim to disentangle the experiences of resilience, discrimination, and mental health with larger, more diverse sample sizes. It may be that both resilience mechanisms and experiences of discrimination vary both across and within racial and ethnic identities which may, in turn, have differential impacts on mental health across time.

Our study has several limitations to note. First, our study sample consisted of individuals who opted into the COPE survey and there was some loss to follow up after the first COPE survey; thus, our findings may not be representative of the United States population as a whole. Second, our study did not ascertain the type of discrimination that an individual perceived (e.g., racial discrimination, gender discrimination) which could potentially differentially impact well-being over time. Third, we were limited in the type of resilience that we could measure through the COPE survey (i.e., resilience to adapt to stressful situations). Future research should focus on other areas of resilience (e.g., social support) when examining the associations between resilience, discrimination, and health among racial and ethnic minorities. Fourth, there are other areas that may have impacted depressive symptoms across time specifically during the COVID-19 pandemic that we were not able to examine in our analyses (e.g., financial strain). Lastly, individuals may differentially experience discrimination based on intersectional identities (e.g., Black gay men); however, we were unable to explore these intersections due to small cell sizes. Future research should consider taking an intersectional focus when examining the impact of resilience on the association between discrimination and health across time using large, diverse samples. Also, there are many different racial/ethnic groups within the categories of Black and Hispanic. It could be that these differences are related to different cultural realities that could lead to difference in the association between resilience and psychological distress. These within-group differences should be examined in future research. Despite these limitations, we provide preliminary evidence suggesting the important role of resilience on mental well-being across time in the face of stress that should be explored in further work.

Our findings suggest that exposure to increased discrimination across the early COVID-19 pandemic negatively impacted mental health over time. However, those who were more resilient in adapting to stress demonstrated lower levels of depressive symptoms in the face of increased discrimination as compared to those who were less resilient across this time period. These findings have several key implications. With respect to research, longitudinal work should focus on examining the mechanisms underpinning the development of healthy resilience as well as resilience mechanisms we were unable to explore here (e.g., social support, emotion regulation). Further, this work should explore potential differences in these mechanisms across cultural contexts and identities. For instance, do resilience mechanisms differ across racial and ethnic identity or socioeconomic status? Having a deeper understanding of these mechanisms, in turn, will inform the creation of culturally tailored and relevant mental health interventions that aim to improve mental health across time and context. Clinicians and mental health professionals should also consider using tools to foster healthy coping mechanisms in the face of stress across a variety of contexts, including exposure to discrimination. For example, cognitive behavioral therapy techniques could consider the role of race/ethnicity-related stressors among racially and ethnically diverse clients (Metzger et al., 2020). Further, more urgent work needs to be done by policymakers and institutions to advocate against racial discrimination. In summary, our study contributes to findings related to the importance of resilience on depressive symptoms within the context of particularly heightened stress in the form of a pandemic and increased racial discrimination. In order to promote mental health, it is imperative that future work be conducted to promote resilience among racially/ethnically diverse populations as well as to curb racial discrimination and the psychological toll of racial discrimination across time.

## Data availability statement

Publicly available datasets were analyzed in this study. This data can be found here: https://allofus.nih.gov/.

## Ethics statement

The studies involving humans were approved by All of Us Institutional Review Board (IRB). The studies were conducted in accordance with the local legislation and institutional requirements. Written informed consent for participation was not required from the participants or the participants’ legal guardians/next of kin in accordance with the national legislation and institutional requirements.

## Author contributions

EW and SC contributed to the conception of the study. ER, CW, and YX performed the statistical analyses and wrote an initial draft of the manuscript. EW wrote the remaining drafts of the manuscript. SC edited the manuscript. All authors contributed to the article and approved the submitted version.
