# Supplementary material for: A national examination of discrimination, resilience, and depressive symptoms during the COVID-19 pandemic: the All of Us Research Program
Source: Front Psychol. 2023 Sep 26;14:1175452. doi: 10.3389/fpsyg.2023.1175452 (PMC10562571; doi:10.3389/fpsyg.2023.1175452)
Supplement: Supplementary file 1 [file Data_Sheet_1.docx]

**Appendix Table 1. Race-stratified regression results**

1. White (N = 45078 )

|  | **Main-effect model** | **Interaction model** |
| --- | --- | --- |
|  | Coef. (SE) | Coef. (SE) |
| Intercept | 7.52 (0.30)*** | 6.75 (0.30)*** |
| Time (survey waves) | 0.05(0.01)*** | 0.05(0.01)*** |
| gender: Male | -0.31(0.03)*** | -0.31(0.03)*** |
| gender: Transgender: /Other | 0.57(0.17)*** | 0.58(0.17)*** |
| sexual: Lesbian or Gay | 0.42(0.08)*** | 0.43(0.08)*** |
| sexual: Bisexual | 0.98(0.08)*** | 1.00(0.08)*** |
| sexual: None | 0.91(0.14)*** | 0.91(0.14)*** |
| Age at baseline | -0.04(0.001)*** | -0.04(0.001)*** |
| income: 25k - 50k | -0.63(0.07)*** | -0.62(0.07)*** |
| income: 50k - 75k | -1.01(0.07)*** | -1.00(0.07)*** |
| income: 75k or greater | -1.07(0.06)*** | -1.06(0.06)*** |
| education: Twelve Or GED | -0.82(0.27)** | -0.71(0.27)** |
| education: College One to Three | -0.78(0.26)** | -0.67(0.26)* |
| education: College graduate | -1.19(0.26)*** | -1.07(0.26)*** |
| education: Advanced degree | -1.20(0.26)*** | -1.09(0.26)*** |
| Resilience (baseline) | -0.23(0.01)*** | -0.19(0.01)*** |
| Loneliness | 0.33(0.004)*** | 0.33(0.004)*** |
| COVID-related impact | 0.28(0.003)*** | 0.28(0.003)**** |
| Social support | -0.005(0.002)** | -0.005(0.002)** |
| Discrimination | 0.21(0.005)*** | 0.48(0.02)*** |
| Discrimination × Resilience (baseline) | - | -0.02(0.001)*** |
| Log Likelihood | -189,571.90 | -189.470.40 |
| Akaike Inf. Crit. | 379,187.80 | 378,986.80 |

(b) Black (*N* = 2705):

|  | **Main-effect model** | **Interaction model** |
| --- | --- | --- |
|  | Coef. (SE) | Coef. (SE) |
| Intercept | 3.48 (0.74) | 2.87 (0.78) |
| Time (survey waves) | -0.004(0.04) | -0.004(0.04) |
| gender: Male | -0.78(0.18)*** | -0.77(0.18)*** |
| gender: Transgender: /Other | -1.20(1.13) | -1.22(1.13) |
| sexual: Lesbian or Gay | 0.25(0.37) | 0.35(0.37) |
| sexual: Bisexual | 1.22(0.39)** | 1.20(0.39)** |
| sexual: None | 2.15(0.71)** | 2.14(0.71)** |
| Age at baseline | -0.04(0.01)*** | -0.05(0.01)*** |
| income: 25k - 50k | -0.75(0.21)*** | -0.74(0.21)*** |
| income: 50k - 75k | -0.43(0.23) | -0.41(0.23) |
| income: 75k or greater | -0.45(0.22)* | -0.44(0.22)* |
| education: Twelve Or GED | -0.10(0.50) | -0.07(0.50) |
| education: College One to Three | -0.03(0.47) | 0.003 (0.47) |
| education: College graduate | -0.54(0.49) | -0.52(0.48) |
| education: Advanced degree | -0.67(0.49) | -0.65(0.49) |
| Resilience (baseline) | -0.13(0.03)*** | -0.09(0.03)** |
| Loneliness | 0.39(0.02)*** | 0.39(0.02)*** |
| COVID-related impact | 0.24(0.01)*** | 0.23(0.01)*** |
| Social support | 0.03(0.01)*** | 0.03(0.01)*** |
| Discrimination | 0.15(0.01)*** | 0.29(0.06)*** |
| Discrimination × Resilience (baseline) | - | -0.01(0.004)** |
| Log Likelihood | -10,392.09 | -10,388.78 |
| Akaike Inf. Crit. | 20,828.18 | 20,823.57 |

^*^p<0.05, ^**^p<0.01, ^***^p<0.001

1. Asian (N=1587)

|  | **Main-effect model** | **Interaction model** |
| --- | --- | --- |
|  | Coef. (SE) | Coef. (SE) |
| Intercept | 5.62 (0.98)*** | 4.94 (1.02)*** |
| Time (survey waves) | 0.13(0.06)* | 0.13(0.06)* |
| gender: Male | -0.55(0.19)** | -0.54(0.19)** |
| gender: Transgender: /Other | 0.83(0.77) | 0.97(0.77) |
| sexual: Lesbian or Gay | 0.14(0.45) | 0.14(0.45) |
| sexual: Bisexual | 2.06(0.43)*** | 2.07(0.43)*** |
| sexual: None | 1.42(0.60)* | 1.38(0.60)* |
| Age at baseline | -0.05(0.01)*** | -0.05(0.01)*** |
| income: 25k - 50k | -0.79(0.38)* | -0.86(0.38)* |
| income: 50k - 75k | -0.70(0.39) | -0.75(0.39) |
| income: 75k or greater | -1.05(0.33)** | -1.10(0.33)*** |
| education: Twelve Or GED | - | - |
| education: College One to Three | -0.68(0.65) | -0.69(0.64) |
| education: College graduate | -1.27(0.60)* | -1.26(0.60)* |
| education: Advanced degree | -1.48(0.60)* | -1.48(0.60)* |
| Resilience (baseline) | -0.20(0,03)*** | -0.15(0.04)*** |
| Loneliness | 0.34(0.02)*** | 0.34(0.02)*** |
| COVID-related impact | 0.30(0.02)*** | 0.30(0.02)*** |
| Social support | 0.02(0.01) | 0.01(0.01) |
| Discrimination | 0.19(0.02)*** | 0.45(0.11)*** |
| Discrimination × Resilience (baseline) | - | -0.02(0.01)* |
| Log Likelihood | -6.319.21 | -6,316.23 |
| Akaike Inf. Crit. | 12,680.41 | 12676.45 |

^*^p<0.05, ^**^p<0.01, ^***^p<0.001

1. Latinx (N=1042)

|  | **Main-effect model** | **Interaction model** |
| --- | --- | --- |
|  | Coef. (SE) | Coef. (SE) |
| Intercept | 7.85 (1.50)*** | 8.35 (1.59)*** |
| Time (survey waves) | -0.01(0.07) | -0.01(0.07) |
| gender: Male | -0.30(0.27) | -0.31(0.27) |
| gender: Transgender: /Other | 2.91(0.88)*** | 2.94(0.88)*** |
| sexual: Lesbian or Gay | 0.93(0.50) | 0.94(0.50) |
| sexual: Bisexual | 0.75(0.49) | 0.76(0.49) |
| sexual: None | 0.90(0.68) | 0.87(0.68) |
| Age at baseline | -0.03(0.01)*** | -0.03(0.01)*** |
| income: 25k - 50k | -0.19(0.42) | -0.20(0.42) |
| income: 50k - 75k | -0.49(0.43) | -0.51(0.43) |
| income: 75k or greater | -0.71(0.39) | -0.71(0.39) |
| education: Twelve Or GED | -1.65(1.10) | -1.71(1.10) |
| education: College One to Three | -1.49(1.04) | -1.52(1.04) |
| education: College graduate | -2.19(1.04)* | -2.23(1.04)* |
| education: Advanced degree | -2.22(1.05)* | -2.26(1.05)* |
| Resilience (baseline) | -0.26(0.05)*** | -0.29(0.06)*** |
| Loneliness | 0.38(0.03)*** | 0.37(0.03)*** |
| COVID-related impact | 0.26(0.02)*** | 0.26(0.02)*** |
| Social support | 0.01(0.01) | 0.01(0.01) |
| Discrimination | 0.18(0.03)*** | 0.06(0.14) |
| Discrimination × Resilience (baseline) | - | 0.01(0.01) |
| Log Likelihood | -4,389.86 | -4,389.42 |
| Akaike Inf. Crit. | 8,823.72 | 8,824.84 |

^*^p<0.05, ^**^p<0.01, ^***^p<0.001

1. More than one Race (N=1403)

|  | **Main-effect model** | **Interaction model** |
| --- | --- | --- |
|  | Coef. (SE) | Coef. (SE) |
| Intercept | 5.46 (2.04)** | 5.08 (2.08)* |
| Time (survey waves) | 0.07(0.07) | 0.07(0.07) |
| gender: Male | -0.26(0.27) | -0.27(0.27) |
| gender: Transgender: /Other | 0.45(0.81) | 0.47(0.81) |
| sexual: Lesbian or Gay | -0.29(0.54) | -0.30(0.54) |
| sexual: Bisexual | 1.54(0.48)** | 1.52(0.48)** |
| sexual: None | -0.07(0.62) | -0.06(0.62) |
| Age at baseline | -0.03(0.01)*** | -0.03(0.01)*** |
| income: 25k - 50k | -0.52(0.45) | -0.51(0.45) |
| income: 50k - 75k | 0.10(0.45) | 0.09(0.45) |
| income: 75k or greater | -0.69(0.40) | -0.68(0.40) |
| education: Twelve Or GED | 0.46(1.79) | 0.44(1.79) |
| education: College One to Three | 1.03(1.75) | 1.07(1.75) |
| education: College graduate | 0.53(1.75) | 0.57(1.750 |
| education: Advanced degree | 0.29(1.75) | 0.32(1.75) |
| Resilience (baseline) | -0.25(0.05)*** | -0.22(0.05)*** |
| Loneliness | 0.38(0.03)*** | 0.38(0.03)*** |
| COVID-related impact | 0.34(0.02)*** | 0.34(0.02)*** |
| Social support | -0.02(0.01) | -0.02(0.01) |
| Discrimination | 0.14(0.03)*** | 0.26(0.14) |
| Discrimination × Resilience (baseline) | - | -0.01(0.01) |
| Log Likelihood | -3,746.36 | -3,745.95 |
| Akaike Inf. Crit. | 7536.71 | 7537.90 |

^*^p<0.05, ^**^p<0.01, ^***^p<0.001

1. Other (N=1403)

|  | **Main-effect model** | **Interaction model** |
| --- | --- | --- |
|  | Coef. (SE) | Coef. (SE) |
| Intercept | 7.19 (2.00)*** | 5.41 (2.05)** |
| Time (survey waves) | -0.06(0.09) | -0.06(0.09) |
| gender: Male | -0.32(0.31) | -0.34(0.30) |
| gender: Transgender: /Other | 0.83(1.62) | 1.10(1.61) |
| sexual: Lesbian or Gay | -0.86(0.65) | -0.89(0.64) |
| sexual: Bisexual | -0.73(0.77) | -0.63(0.76) |
| sexual: None | 0.62(0.85) | 0.62(0.84) |
| Age at baseline | -0.04(0.01)*** | -0.04(0.01)*** |
| income: 25k - 50k | 0.67(0.49) | -0.45(0.51) |
| income: 50k - 75k | -0.48(0.52) | -0.20(0.45) |
| income: 75k or greater | -0.20(0.45) | 0.15(1.72) |
| education: Twelve Or GED | 0.19(1.73) | -0.49(1.65) |
| education: College One to Three | -0.58(1.66) | -0.85(1.66) |
| education: College graduate | -0.99(1.67) | -1.14(1.66) |
| education: Advanced degree | -1.25(1.68) | -0.15(0.06)* |
| Resilience (baseline) | -0.25(0.05)*** | 0.42(0,03)*** |
| Loneliness | 0.42(0.03)*** | 0.27(0.03)*** |
| COVID-related impact | 0.27(0.03)*** | -0.004(0.01) |
| Social support | -0.01(0.01) | 0.65(0.03)*** |
| Discrimination | 0.22(0.03)*** | 0.64 (0.13)*** |
| Discrimination × Resilience (baseline) | - | -0.45(0.51) |
| Log Likelihood | -2,628.51 | -2,622.13 |
| Akaike Inf. Crit. | 5,301.01 | 5,290.26 |

^*^p<0.05, ^**^p<0.01, ^***^p<0.001

**Appendix Table 2. Test of homogeneity of effects across model stratification**

(a) The coefficients of all race-stratified models next to each other:

| Variables | Coef.(SE) | | | |  |  |  |  |
| --- | --- | --- | --- | --- | --- | --- | --- | --- |
|  | White | Black | Asian |  | | |  |  |
| Time | 0.05*** | -0.004 | 0.13* | | |  | | |
|  | (-0.01) | (0.04) | (0.06) | |  | | |  |
| Discrimination | 0.48*** | 0.29*** | 0.45*** | |  | | |  |
|  | (0.02) | (0.06) | (0.11) | |  | | |  |
| Discrimination × Resilience | -0.02*** | -0.01*** | -0.02* | |  | | |  |
|  | (0.001) | (0.004) | (0.01) | |  | | |  |
| Constant | 6.75*** | 2.87*** | 4.94*** | |  | | |  |
|  | (0.30) | (0.78) | (1.02) | |  | | |  |
| *N* | 45078 | 2750 | 1587 | |  | | |  |

^*^p<0.05, ^**^p<0.01, ^***^p<0.001

(b) Contrasts for the main effect of discrimination on PHQ9 stratified by Race

| Comparison | Estimate (SE) | p-value |
| --- | --- | --- |
| White – Black | 0.07 (0.01) | <0.001 |
| White – Asian | 0.01 (0.02) | 0.97 |
| Asian – Black | 0.06 (0.02) | 0.07 |

(c) Contrasts for the main effect of discrimination on PHQ9 by Resilience

| Comparison | Estimate (SE) | p-value |
| --- | --- | --- |
| 12.3-15 | 0.05 (0.003) | <0.001 |
| 12.3-17.7 | 0.09 (0.006) | <0.001 |
| 15- 17.7 | 0.05 (0.003) | 0.004 |

Where 12.3 represents <12.3, 15 represents 12.3-17.7, and 17.7 represents >17.7 resilience score

(d) Contrasts for the main effect of the interaction between discrimination and resilience on PHQ9 stratified by Race

| Resilience Score: |  | <12.3 | |  | 12.3-17.7 | |  |  | >17.7 |
| --- | --- | --- | --- | --- | --- | --- | --- | --- | --- |
| Comparison | Estimate (SE) | | p-value |  | Estimate (SE) | | p-value | Estimate (SE) | p-value |
| White – Black | 0.08 (0.02) | | <0.001 |  | 0.05 (0.01) | 0.001 | | 0.02 (0.02) | 0.73 |
| White – Asian | 0.004 (0.03) | | 0.99 |  | -0.01(0.02) | 0.95 | | -0.02 (0.03) | 0.79 |
| Asian – Black | 0.07 (0.03) | | 0.06 |  | 0.06 (0.02) | 0.06 | | 0.04 (0.03) | 0.50 |
